# Retraction: The Efficacy of Artificial Intelligence in the Detection and Management of Atrial Fibrillation 

**DOI:** 10.7759/cureus.r226

**Published:** 2026-04-16

**Authors:** Apurva Popat, Sweta Yadav, Jacob Obholz, Elliot A Hwang, Ateeq U Rehman, Param Sharma

**Affiliations:** 1 Internal Medicine, Marshfield Clinic Health System, Marshfield, USA; 2 Cardiology, Marshfield Clinic Health System, Marshfield, USA

The Editor-in-Chief has retracted this article due to significant inaccuracies in the characterization of cited references. Müller et al., 2023 [40] and Xiong et al., 2022 [56] are described as involving artificial intelligence and predictive modeling; however, both studies instead focus on ablation index and do not contain the reported analyses. These misrepresentations affect the validity and credibility of the manuscript’s content and conclusions. The findings are considered unreliable and the article is therefore retracted. The authors agree with the decision to retract.

